# Continuous Anchor-Confidence-Weighted UWB/IMU Localization for Unmanned Ground Vehicles in Structured Indoor Environments

**DOI:** 10.3390/s26165215

**Published:** 2026-08-17

**Authors:** Yufei Yang, Wei Liu

**Affiliations:** School of Automation, Jiangsu University of Science and Technology, Zhenjiang 212003, China; 232241821230@stu.just.edu.cn

**Keywords:** indoor localization, unmanned ground vehicle, ultra-wideband, multi-sensor fusion

## Abstract

**Highlights:**

**What are the main findings?**

- UWB/IMU localization in structured indoor environments is limited by position-dependent anchor visibility and mixed LOS/NLOS ranging.
- A virtual-iteration-derived soft continuous confidence encoding and weighting method is developed for WLS and AKF localization.

**What are the implications of the main findings?**

- Soft WLS reduces the calibration-point RMSE from 0.490 m to 0.343 m relative to Hard WLS.
- Soft-weighted AKF lowers the mean weighted range-consistency residual by 42.4% relative to Hard-weighted EKF.

**Abstract:**

In Global Navigation Satellite System (GNSS)-denied indoor environments, ultra-wideband (UWB) localization of unmanned ground vehicles (UGVs) is challenged by position-dependent anchor visibility and mixed line-of-sight (LOS)/non-line-of-sight (NLOS) ranging. This study proposes a soft continuous confidence weighting method within an adaptive Kalman filter (AKF)-based UWB/inertial measurement unit (IMU) localization framework. The vehicle model uses motor pulse increments and IMU yaw-rate measurements as inputs and outputs vehicle position and heading estimates. Virtual forward–backward iteration converts inconsistencies between the current UWB ranges and tag–anchor geometry into terminal virtual-anchor displacements. A half-Gaussian function then maps each displacement to a continuous confidence coefficient. The resulting coefficients are incorporated into weighted least-squares (WLS) and AKF localization, while the UWB measurement-noise covariance is adaptively updated using the range innovations. The proposed method was evaluated through static calibration and dynamic localization experiments. These experiments compared soft and hard weighting schemes and assessed the contribution of AKF fusion. These results indicate that the method proposed in this study improves localization accuracy, robustness, and temporal continuity under position-dependent anchor visibility and mixed LOS/NLOS conditions.

## 1. Introduction

Unmanned ground vehicles (UGVs) are increasingly deployed in intelligent logistics, infrastructure inspection, search and rescue, and other hazardous or labor-intensive operations [1,2]. Continuous and reliable localization is essential for autonomous navigation in these applications. In Global Navigation Satellite System (GNSS)-denied indoor environments, candidate solutions include inertial navigation, visual simultaneous localization and mapping (SLAM), light detection and ranging (LiDAR)-inertial odometry, and radio-frequency positioning [3,4,5]. Radio-frequency approaches commonly use Wi-Fi, Bluetooth, or ultra-wideband (UWB). Wi-Fi and Bluetooth can reuse existing access points or inexpensive beacons, supporting low-cost and scalable deployment [3]. However, their received signal strength (RSS) measurements are sensitive to multipath and signal fluctuations, and the resulting RSS fingerprints are environment-dependent [3]. Wi-Fi fine time measurement improves ranging precision, but UWB generally provides finer line-of-sight ranging in industrial environments [6]. UWB achieves this precision through direct wideband time-of-flight (ToF) measurements [7,8].

Visible light communication (VLC)-based positioning, commonly termed visible light positioning (VLP), forms a distinct optical positioning modality [9]. Xu et al. [10] optimized VLC fingerprint weights using a meta-heuristic method and achieved a 3.11 cm mean experimental error at a 50% random selection rate. VLP can reuse light-emitting diode (LED) luminaires and achieve centimeter-order accuracy without radio-frequency interference [9,10]. However, practical performance depends on illumination coverage, receiver orientation, and a sufficient number of visible LED links [9]. Ambient light, optical blockage, and luminaire retrofit or calibration can also reduce robustness and increase deployment effort [9]. Compared with VLP, UWB does not rely on illumination infrastructure and is insensitive to ambient-light variation [6,7,8,9]. It can also provide wider continuous coverage across connected indoor areas when anchors are appropriately placed [6,7,8,9]. These advantages favor UWB for continuous UGV localization in structured corridors, although dedicated anchors must be installed.

UWB has been integrated into indoor mobile-robot SLAM [11] and used for relative guide localization in UGV follow-me operation [12]. It has also supported low-cost relative UGV localization [13] and distributed cooperative localization for unmanned systems [14]. Xu et al. [15] proposed an anchor-free unmanned aerial vehicle (UAV)–UGV collaborative localization method that fuses visual–inertial odometry (VIO) with UWB measurements. These studies demonstrate the broad application potential of UWB in robotic localization.

Despite these advantages, UWB ranging in indoor environments is susceptible to non-line-of-sight (NLOS) propagation and multipath interference. Walls, corners, and fixed structures may obstruct the direct propagation path and introduce positive ranging biases through reflection, diffraction, or penetration. Changes in propagation conditions may also produce abnormal measurements that degrade the subsequent state correction [16,17]. For the UGV platform considered in this study, encoder-pulse-derived forward velocity and inertial measurement unit (IMU) yaw rate are used for motion prediction, while UWB ranges provide geometric information for position correction. Although the integration of these measurements improves localization continuity, sensor fusion alone cannot determine the reliability of an individual UWB range before it contributes to the adaptive Kalman filter (AKF) update.

Existing studies relevant to this research problem can be broadly categorized into three streams. The first focuses on fusion architecture. Loosely coupled schemes fuse UWB-derived positions, but their accuracy depends on the upstream UWB positioning solution. Tightly coupled schemes directly incorporate individual UWB ranges and retain range-level constraints. They can therefore down-weight unreliable ranges or reject severe outliers when only a few reliable anchors are available. Mi et al. [18] developed a constrained microelectromechanical system (MEMS)-based inertial navigation system (INS)/UWB method with Allan-variance-assisted error modeling and adaptive EKF correction. Lin et al. [19] proposed an enhanced UGV localization framework using tightly coupled IMU/UWB/barometer integration (EULF-IUB), which combines robust range smoothing, horizontal localization, and barometric height correction. These works show the benefit of tightly coupled fusion, but range reliability still needs to be handled explicitly in complex indoor environments.

The second improves tightly coupled positioning by adding estimator-level or sensor-error compensation mechanisms. Zhu et al. [20] developed an error-state Kalman filter (ESKF) with adaptive measurement-noise covariance for UWB/IMU-based mine-loader positioning. Temperature-drift compensation and zero-velocity detection further reduce IMU errors. Wang et al. [21] proposed a cubature information filter with variational Bayesian noise estimation for time-varying line-of-sight (LOS) and NLOS conditions. These methods improve estimator robustness and sensor-error handling. However, they do not exploit static environmental geometry to assess the structural visibility of individual UWB links.

The third focuses on unreliable-range identification, NLOS mitigation, and geometric consistency. Sun et al. [16] combined UWB signal statistics with IMU/odometer motion consistency for two-stage NLOS detection and estimator selection. H. Zhang et al. [17] constructed a spatial LOS/NLOS information map in which each grid point is assigned a binary anchor-state code. At an estimated position, the codes of neighboring grid points are combined using a logical AND operation to obtain the corresponding anchor mask. T. Zhang et al. [22] proposed a tightly coupled UWB–LiDAR–inertial estimator with multi-epoch outlier rejection (MR-ULINS). It uses a multi-epoch test based on random sample consensus (RANSAC) to reject UWB outliers and estimate range bias and scale online. Sui et al. [23] combined NLOS-angle discrimination with map constraints to reduce the effect of degraded UWB measurements. Lu et al. [24] proposed a virtual-iteration-based back-end optimizer that jointly refines positions and ranging errors from virtual-anchor displacements. These studies provide useful mechanisms for NLOS mitigation and geometric consistency evaluation. However, binary structural visibility and continuous geometric consistency have rarely been evaluated as alternative per-range reliability representations under a matched localization framework.

Building upon spatial-prior visibility modeling [17] and virtual-iteration-based geometric-consistency analysis [24], this paper develops a UGV localization framework using soft continuous confidence encoding and weighting. The confidence coefficients at the current vehicle position are obtained directly through virtual iteration rather than from neighboring points. For ease of presentation, the terms hard encoding and hard weighting refer to the hard binary scheme in [17], whereas soft encoding and soft weighting refer to the proposed soft continuous scheme in this paper. The main contributions of this study are summarized as follows:

This paper proposes a soft continuous confidence encoding and weighting method that enables more reasonable and precise allocation of individual anchor contributions to localization.

The soft continuous confidence encoding can be obtained directly for the current vehicle position using the virtual-iteration method.

An AKF is combined with a vehicle model that uses motor pulse increments and yaw-rate measurements as inputs and provides vehicle position and heading as outputs.

The remainder of this paper is organized as follows. Section 2 introduces the localization scenario and problem formulation and establishes the UGV motion and UWB ranging models. Section 3 describes how the soft continuous confidence encoding is obtained and integrated into the weighted least-squares (WLS) and adaptive Kalman filter (AKF) frameworks. Section 4 reports the surveyed-point calibration and dynamic UGV localization experiments. Section 5 concludes the paper.

## 2. Problem Description and Mathematical Formulation

### 2.1. Indoor UGV Localization Scenario and UWB Link Reliability

In practical indoor UGV applications, such as warehouse logistics, infrastructure inspection, and underground utility inspection, vehicles often operate in environments containing walls, corners, and fixed structural obstructions. These structures make the direct propagation path between a vehicle-mounted UWB tag and fixed anchors dependent on the vehicle position. Consequently, the UWB measurements collected during vehicle motion may contain both LOS ranges and NLOS ranges with positive bias.

The LOS/NLOS condition of a UWB ranging link is not a fixed property of an anchor, but a position-dependent link state determined by the vehicle location and surrounding geometry. As the UGV moves through different passage segments, an anchor that is blocked in one segment may become visible in another segment, while previously visible anchors may become blocked after the vehicle passes a corner. This position-dependent visibility makes it difficult to determine, at each sampling instant, which ranging measurements can be used as reliable observations for sensor fusion. If structurally blocked links are directly used in the AKF update, their positive range biases may distort the state correction. Therefore, the reliability of each UWB range must be determined at each sampling instant before its contribution to the AKF correction is specified.

**Figure 1:**
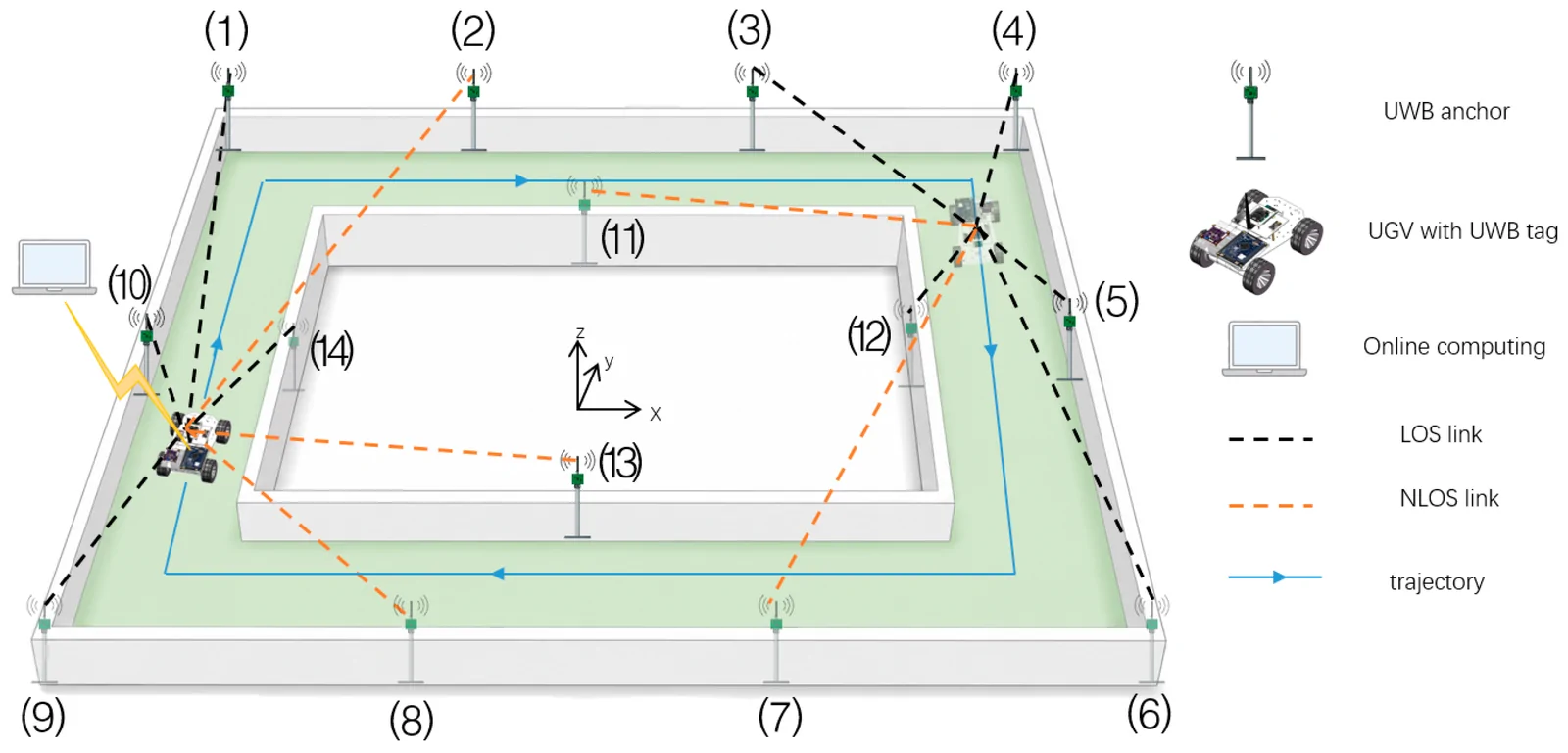
Representative indoor layout illustrating position-dependent UWB LOS/NLOS links.

To illustrate the above position-dependent visibility problem, Figure 1 presents a representative indoor layout with structural obstructions. Fixed UWB anchors are deployed along the passage boundaries and near the corner regions, while the UGV carries a UWB tag and moves along the predefined loop-shaped trajectory. When the vehicle is located in the left corridor segment, nearby anchors such as (1), (9), (10), and (14) can form LOS links, whereas many anchors in the right and inner regions are blocked by walls or corners. When the vehicle moves to the right corridor segment, anchors such as (3), (4), (5), (6), and (12) become more favorable LOS candidates, while several anchors on the left side may become unavailable. This position-dependent change indicates that the reliability of an anchor link is not fixed during vehicle motion. Consequently, the UWB ranges should not be treated as equally reliable at every sampling instant. The detailed hardware platform and implementation workflow are described later in Section 4.

### 2.2. UGV Motion and UWB Ranging Models

The UGV is modeled as a planar vehicle moving on the horizontal plane. Considering that the onboard motion measurements are acquired at discrete sampling instants, the vehicle state at sampling instant k is defined as

(1) $$X_{k}={\left[x_{k},y_{k},\theta_{k}\right]}^{T}$$

where $x_{k}$. and $y_{k}$ denote the planar position coordinates, and $\theta_{k}$ is the heading angle.

At sampling instant k, the motion input is defined as

(2) $$u_{k}={\left[v_{k},\omega_{k}\right]}^{T}$$

where $v_{k}$ denotes the forward velocity and $\omega_{k}$ denotes the yaw rate. In the implementation, $v_{k}$ is calculated from the motor pulse increments, as detailed in Section 4.1. The corresponding disturbance vector is denoted as

(3) $$\xi_{k}={\left[\xi_{v,k},\xi_{\omega ,k}\right]}^{T}$$

where $\xi_{v,k}$ and $\xi_{\omega ,k}$ represent the forward-velocity disturbance and yaw-rate disturbance.

Using a first-order discretization with sampling interval Δt, the discrete planar kinematic model is expressed as

(4) $$\begin{array}{l} x_{k+1}=x_{k}+(v_{k}+\xi_{v,k})\cos\theta_{k}\Delta t\\ y_{k+1}=y_{k}+(v_{k}+\xi_{v,k})\sin\theta_{k}\Delta t\\ \theta_{k+1}=\theta_{k}+(\omega_{k}+\xi_{\omega ,k})\Delta t \end{array}$$

Let the coordinate of fixed UWB anchor i be

(5) $$a_{i}={\left[x_{a_{i}},y_{a_{i}}\right]}^{T},\;i=1,2,\ldots ,N$$

where N is the total number of fixed UWB anchors deployed in the localization environment. At sampling instant k, the true but unknown planar position of the vehicle-mounted UWB tag is denoted as

(6) $$p_{k}={\left[x_{k},y_{k}\right]}^{T}$$

Since the UWB tag is rigidly mounted on the UGV and the installation offset between the tag and the vehicle reference point is neglected or calibrated, $p_{k}$ is used to represent the planar position of the vehicle in this study.

The UWB range is obtained using double-sided two-way ranging (DS-TWR) based on ToF estimation [25]. The ranging measurement between the vehicle-mounted tag and anchor i is modeled as

(7) $$d_{i,k}=\|p_{k}-a_{i}{\|}_{2}+\varepsilon_{i,k}$$

where $d_{i,k}$ is the measured range and $\varepsilon_{i,k}$ denotes the residual ranging error. Under mixed LOS/NLOS propagation conditions, $\varepsilon_{i,k}$ can be written as

(8) $$\varepsilon_{i,k}=\eta_{i,k}+b_{i,k}$$

where $\eta_{i,k}$ is the random ranging noise and $b_{i,k}\geq 0$ represents the positive bias caused by NLOS propagation. Under LOS propagation $b_{i,k}$ is assumed negligible, and the measurement is mainly determined by the geometric distance between the tag and the anchor. Under NLOS propagation, the direct path is blocked, and the measured range is usually larger than the true geometric distance.

### 2.3. Localization Problem Formulation

The localization problem considered in this study is to estimate the planar vehicle state by fusing encoder-derived forward velocity, IMU-measured yaw rate, and UWB range measurements under mixed LOS/NLOS propagation conditions. At each sampling instant k, the UWB range vector collected from the fixed anchors provides position-related observations, whereas the encoder and IMU measurements provide motion constraints for AKF prediction.

(9) $$d_{k}={\left[d_{1,k},d_{2,k},\ldots ,d_{N,k}\right]}^{T}$$

Given the range vector $d_{k}$, the motion input $u_{k}$, the known anchor coordinates, and the static geometric information of the environment, the objective is to obtain a stable estimate of the vehicle position while reducing the influence of NLOS-contaminated measurements.

This problem requires not only multi-sensor state estimation but also measurement-level reliability assignment before AKF correction. Each UWB range should be associated with a confidence value that reflects both structural visibility and geometric consistency. This confidence value determines the contribution of the corresponding range measurement to the AKF update.

In this study, two sources of UWB ranging degradation are considered. The first is structural NLOS caused by static walls, corners, and fixed obstacles. This type of degradation is spatially repeatable and can be described using the geometric information of the environment. The second is residual ranging inconsistency caused by temporary occlusion, multipath propagation, or local environmental changes that cannot be fully represented by the static map.

Accordingly, Section 3 presents a method for deriving a soft continuous confidence encoding for each anchor at the current estimated position and using these encodings to weight the corresponding UWB range measurements.

## 3. Localization Method

### 3.1. Scene Discretization and Anchor-Wise Structural-Visibility Encoding

**Figure 2:**
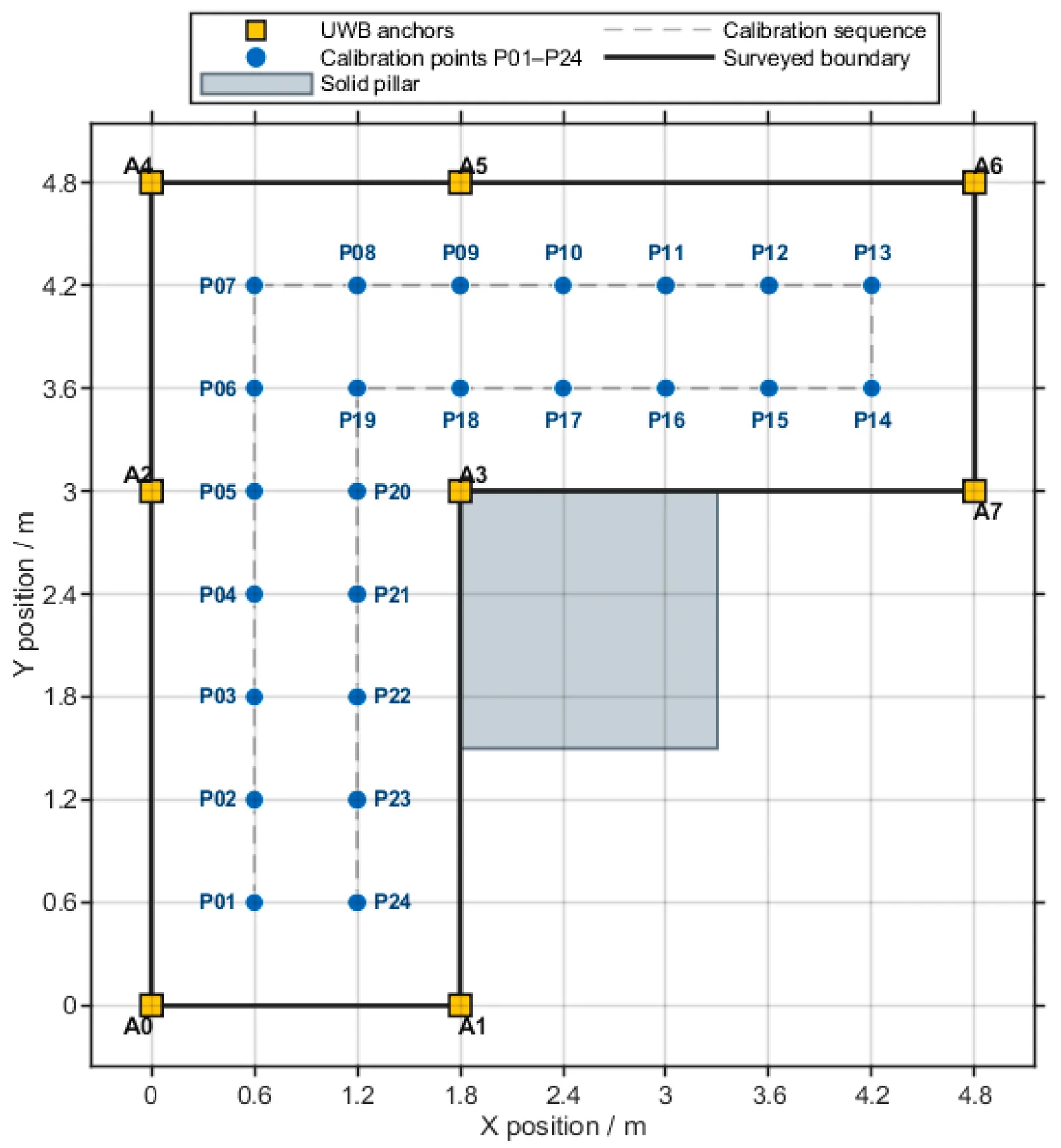
Grid-based layout of the surveyed indoor scene for structural-visibility evaluation.

To assess the spatial variation in UWB link visibility across the indoor scene, the traversable region Ω is first discretized into a set of grid points. Walls and fixed solid obstacles are represented by the obstacle set O. The grid-point set is defined as

(10) $$G={\left\{g_{j}\right\}}_{j=1}^{M},\;g_{j}={\left[x_{j},y_{j}\right]}^{T}$$

where M is the number of grid points. The anchor coordinates, obstacle boundaries, and grid points are registered in the same two-dimensional coordinate system. These grid points provide spatial samples for evaluating the structural visibility of individual tag–anchor links. The resulting grid-based representation of the surveyed scene is illustrated in Figure 2.

Following the LOS/NLOS mapping principle in [17], the structural visibility between each grid point $g_{j}$ and anchor $a_{i}$ is determined by testing whether their connecting line segment intersects the obstacle set. The corresponding binary state is defined as

(11) $$s_{i,j}=\left\{\begin{array}{ll} 1, & \bar{g_{j}a_{i}}\cap O=\emptyset ,\\ 0, & \bar{g_{j}a_{i}}\cap O\neq \emptyset , \end{array}\right.\;i=1,2,\ldots ,N,\;j=1,2,\ldots ,M$$

A value of $s_{i,j}=1$ indicates that the link is structurally visible with respect to the known fixed obstacles. Conversely, $s_{i,j}=0$ indicates that the direct propagation path is structurally blocked. Here, the LOS/NLOS classification describes map-derived structural visibility. It does not account for temporary occlusion, multipath propagation, or other ranging disturbances not represented by the geometric map.

After all grid–anchor pairs have been evaluated, the binary states are stored in the spatial-prior matrix:

(12) $$S=[s_{i,j}]\in {\left\{0,1\right\}}^{N\times M}$$

Each column of S contains the structural-visibility states of all anchors at one grid point. Thus, for the eight-anchor deployment considered in this study, each grid point is associated with an eight-entry anchor-wise code. This code provides a compact representation of the structural ranging conditions at the corresponding location.

The anchor-wise code associated with grid point $g_{j}$ is incorporated directly into the weighted least-squares (WLS) objective. The resulting position estimate is given by

(13) $${\hat{p}}_{k}^{\mathrm{WLS}}=\underset{p}{\arg\min}\sum_{i=1}^{N}s_{i,j}{\left(\|p-a_{i}{\|}_{2}-d_{i,k}\right)}^{2}$$

Accordingly, measurements from structurally visible links contribute to the WLS objective, whereas structurally blocked links do not contribute because their coefficients are zero. A well-constrained planar solution requires a sufficient number of retained anchors with suitable geometry.

During vehicle motion, however, the tag position does not generally coincide with a predefined grid point. An anchor-wise code is nevertheless required to describe the reliability of the N ranging links at the current vehicle position. Such a code can be expressed as either a hard binary representation or a soft continuous representation. The hard code produces a retain-or-discard decision, whereas the soft code can preserve graded differences in the consistency of individual range measurements. Section 3.2 therefore introduces a soft anchor-wise code derived from virtual-iteration-based geometric consistency.

### 3.2. Virtual-Iteration-Based Geometric-Consistency Confidence Modeling

To further obtain a soft and continuous confidence code, the virtual forward–backward iteration in ref. [24] is adopted to evaluate the geometric consistency between the current UWB ranges and the tag–anchor geometry. A direct range residual is affected jointly by tag-position initialization error, random ranging noise, and NLOS-induced bias. It is therefore not mapped directly to the confidence code. Instead, the virtual iteration converts the range inconsistencies into virtual-anchor displacements, which are subsequently mapped to continuous confidence coefficients.

At sampling instant k, the virtual tag is initialized using an available tag-position estimate, denoted by $p_{k}^{\mathrm{init}}$, while the virtual anchors are initialized at their physical coordinates:

(14) $${\tilde{p}}_{k}^{\left(0\right)}=p_{k}^{\mathrm{init}},{\tilde{a}}_{i,k}^{\left(0\right)}=a_{i}\;i=1,\ldots ,N$$

The virtual forward–backward mechanism in ref. [24] is adopted in this study. To facilitate the experimental analysis, all UWB anchors were installed at the same height and treated as lying in a common horizontal plane. At iteration t, the reverse update is first performed by fixing the virtual tag position obtained in the previous iteration, ${\tilde{p}}_{k}^{\left(t-1\right)}$. For anchor i, the virtual tag position is used as the first reference node:

(15) $${\tilde{q}}_{i,0,k}^{\left(t\right)}=\;{\tilde{p}}_{k}^{\left(t-1\right)}$$

Auxiliary reference nodes are then introduced following [24]. The corresponding distance constraints are defined as

(16) $$\rho_{i,0,k}^{\left(t\right)}=d_{i,k},\;\rho_{i,j,k}^{\left(t\right)}={\left\|\;{\tilde{p}}_{k}^{\left(t-1\right)}-{\tilde{a}}_{i,k}^{\left(t-1\right)}\right\|}_{2},\;j=1,2,3$$

The virtual anchor position is subsequently updated by solving

(17) $${\tilde{a}}_{i,k}^{\left(t\right)}=\underset{a}{\arg\min}\sum_{j=0}^{3}{\left({\left\|a-{\tilde{q}}_{i,j,k}^{\left(t\right)}\right\|}_{2}^{2}-{\left(\rho_{i,j,k}^{\left(t\right)}\right)}^{2}\right)}^{2}$$

After the reverse update, the virtual anchors are fixed, and the virtual tag position is updated through forward localization:

(18) $${\tilde{p}}_{k}^{\left(t\right)}=\underset{p}{\arg\min}\sum_{i=1}^{N}{\left[{\left\|p-\;{\tilde{a}}_{i,k}^{\left(t\right)}\right\|}_{2}^{2}-d_{i,k}^{2}\right]}^{2}$$

One reverse update followed by one forward update constitutes a complete virtual iteration. Following ref. [24], the iteration terminates when the maximum position change of the virtual tag and virtual anchors does not exceed 0.1 m or when the iteration count reaches 20. Under the local approximation used in the same study, let $J_{\mathrm{VI}}^{*}$ denote the linearized error-state transition matrix for a complete reverse–forward update. A sufficient condition for local convergence is

(19) $$\mathrm{spr}(J_{\mathrm{VI}}^{*})=\underset{\lambda \in \mathrm{spec}(J_{\mathrm{VI}}^{*})}{\max}|\lambda |<1$$

Here, spec(·) denotes the set of eigenvalues, and spr(·) denotes the spectral radius. The detailed error-propagation and convergence analyses are provided in ref. [24]. This condition is local and does not establish global convergence.

**Figure 3:**
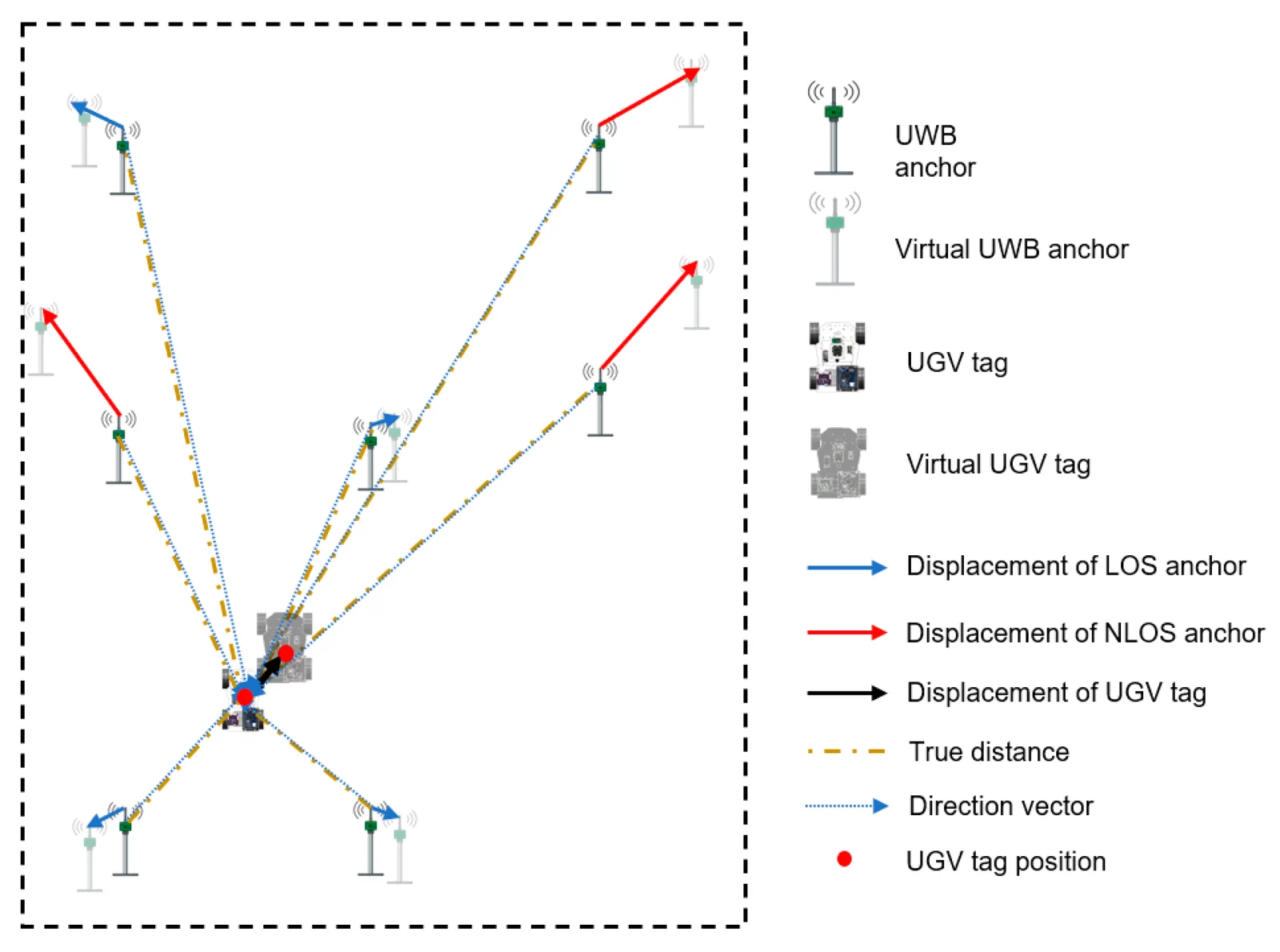
Virtual-iteration-based representation of anchor-wise geometric consistency.

The terminal virtual tag and anchor positions are denoted by ${\tilde{p}}_{k}^{*}$ and ${\tilde{a}}_{i,k}^{*}$, respectively. Their displacements are virtual quantities and do not modify the input tag position or the physical anchor coordinates. As illustrated in Figure 3, the displacement of each virtual anchor is used to characterize the geometric consistency of its associated UWB range.

For anchor i, the terminal virtual-anchor displacement is defined as

(20) $$d_{i,k}^{\mathrm{VI}}={\left\|\;{\tilde{a}}_{i,k}^{*}-a_{i}\right\|}_{2}$$

A small $d_{i,k}^{\mathrm{VI}}$ indicates that only a minor displacement from the physical anchor coordinate is required to reconcile the corresponding range with the current geometry. Conversely, a large displacement indicates a stronger geometric inconsistency. The displacement is therefore treated as a continuous consistency indicator rather than a binary LOS/NLOS classification.

The virtual-anchor displacement is mapped to a soft anchor-trust coefficient using a half-Gaussian function:

(21) $$\gamma_{i,k}=\exp\left[-\frac{1}{2}{\left(\frac{d_{i,k}^{\mathrm{VI}}}{\sigma_{\mathrm{VI}}}\right)}^{2}\right],\;0<\gamma_{i,k}\leq 1$$

Here, $\sigma_{\mathrm{VI}}$ denotes the half-Gaussian decay scale. The trust coefficient equals one when the terminal virtual anchor coincides with its physical coordinate and decreases continuously as the displacement increases.

The resulting soft confidence code is expressed as

(22) $$\gamma_{k}={\left[\gamma_{1,k},\gamma_{2,k},\ldots ,\gamma_{N,k}\right]}^{T}$$

For the eight-anchor deployment considered in this study, $\gamma_{k}$ is an eight-entry column vector. It has the same anchor-wise dimensionality as the hard binary code in Section 3.1 while preserving continuous differences in the geometric consistency of the UWB ranges.

### 3.3. AKF Update with Soft Confidence Weighting

After the soft confidence code is obtained in Section 3.2, it is incorporated into the AKF update to regulate the contribution of individual UWB ranges. The motor pulse increments and IMU-measured yaw rate are used in the prediction step, while the UWB measurements correct the predicted state. During the measurement update, the measurement-noise covariance is adaptively estimated from the range innovations.

According to the discrete planar kinematic model in Section 2.2, the state transition model is written as

(23) $$X_{k}=f(X_{k-1},u_{k-1},\xi_{k-1})$$

For the UWB ranges used in the current update, the observation model is written as

(24) $$Z_{k}=\left[\begin{array}{c} d_{i_{1},k}\\ d_{i_{2},k}\\ \vdots \\ d_{i_{m},k} \end{array}\right]=\left[\begin{array}{c} \sqrt{{\left(x_{k}-x_{a_{i_{1}}}\right)}^{2}+{\left(y_{k}-y_{a_{i_{1}}}\right)}^{2}}\\ \sqrt{{\left(x_{k}-x_{a_{i_{2}}}\right)}^{2}+{\left(y_{k}-y_{a_{i_{2}}}\right)}^{2}}\\ \vdots \\ \sqrt{{\left(x_{k}-x_{a_{i_{m}}}\right)}^{2}+{\left(y_{k}-y_{a_{i_{m}}}\right)}^{2}} \end{array}\right]+\left[\begin{array}{c} \varepsilon_{i_{1},k}\\ \varepsilon_{i_{2},k}\\ \vdots \\ \varepsilon_{i_{m},k} \end{array}\right]$$

where m is the number of valid UWB ranges included in the observation vector at sampling instant k.

The predicted state is obtained by setting the noise term in the state equation to zero:

(25) $${\hat{X}}_{k|k-1}=f({\hat{X}}_{k-1|k-1},u_{k-1},0)$$

The predicted covariance matrix is propagated as

(26) $$P_{k|k-1}=F_{x,k-1}P_{k-1|k-1}F_{x,k-1}^{T}+F_{u,k-1}Q_{u,k-1}F_{u,k-1}^{T}$$

where $F_{x,k-1}$ and $F_{u,k-1}$ are the Jacobian matrices of the state transition function with respect to the state and the motion input, respectively, and $Q_{u,k-1}$ is the covariance matrix associated with the motion-input uncertainty.

The nonlinear observation function corresponding to the current observation vector is denoted as $h(X_{k})$, and its Jacobian evaluated at the predicted state is

(27) $$H_{k}={\left.\frac{\partial h(X_{k})}{\partial X_{k}}\right|}_{{\hat{X}}_{k|k-1}}$$

To account for time-varying UWB ranging uncertainty, the diagonal elements of the measurement-noise covariance matrix are adaptively estimated. For each valid UWB range, the instantaneous variance estimate is calculated as

(28) $${\hat{R}}_{i_{j},k}^{\mathrm{inst}}=\max\left\{{\left[Z_{k}-h\left({\hat{X}}_{k|k-1}\right)\right]}_{j}^{2}-{\left[H_{k}P_{k|k-1}H_{k}^{T}\right]}_{jj},0\right\}$$

The measurement-noise variance is then updated using a forgetting factor:

(29) $$R_{i_{j},k}={\mathrm{sat}}_{\left[R_{\min},R_{\max}\right]}\left[\beta R_{i_{j},k-1}+(1-\beta ){\hat{R}}_{i_{j},k}^{\mathrm{inst}}\right],\;R_{k}=\mathrm{diag}\left(R_{i_{1},k},\ldots ,R_{i_{m},k}\right)$$

Here, $i_{j}$ identifies the anchor associated with the corresponding entry of the current observation vector, β is the forgetting factor, $R_{\min}$ and $R_{\max}$ are the lower and upper variance bounds, respectively. The operator ${\mathrm{sat}}_{\left[R_{\min},R_{\max}\right]}(\cdot )$ limits the updated variance to the specified interval. Using the adaptively updated $R_{k}$, the Kalman gain is calculated as

(30) $$K_{k}=P_{k|k-1}{H_{k}}^{T}{\left[H_{k}P_{k|k-1}{H_{k}}^{T}+R_{k}\right]}^{-1}$$

Because $R_{k}$ is updated online rather than held fixed, the resulting estimator constitutes an AKF.

Let $\gamma_{k}$ contain the entries of the soft confidence code corresponding to the UWB ranges in $Z_{k}$. The confidence-weighted gain is defined as

(31) $${\tilde{K}}_{k}=K_{k}\mathrm{diag}(\gamma_{k})$$

where $\mathrm{diag}(\gamma_{k})$ is the diagonal matrix formed by the corresponding confidence coefficients. This operation scales each column of $K_{k}$ according to the confidence assigned to the associated UWB range. Consequently, geometrically consistent ranges have greater influence on the AKF correction, whereas ranges with lower confidence contribute less.

The posterior state is then updated as

(32) $${\hat{X}}_{k|k}={\hat{X}}_{k|k-1}+{\tilde{K}}_{k}\left[Z_{k}-h({\hat{X}}_{k|k-1})\right]$$

and the posterior covariance matrix is updated as

(33) $$P_{k|k}=(I-{\tilde{K}}_{k}H_{k})P_{k|k-1}{\left(I-{\tilde{K}}_{k}H_{k}\right)}^{T}+{\tilde{K}}_{k}R_{k}{\tilde{K}}_{k}^{T}$$

After the update, the position component of ${\hat{X}}_{k|k}$ is taken as the localization result at time k.

## 4. Experimental Validation

To validate the proposed soft continuous confidence encoding and weighting framework, this section presents the experimental platform, implementation workflow, and comparative evaluations. Section 4.1 describes the UGV platform and hardware configuration, while Section 4.2 presents the software implementation and evaluation flow. Section 4.3 then reports the experimental evaluation of the proposed method. Section 4.3.1 uses calibration-site experiments to compare Hard WLS with Soft WLS and select the confidence-mapping scale. Section 4.3.2 evaluates the contribution of AKF by comparing localization with and without AKF. Section 4.3.3 compares Soft-weighted AKF with Hard-weighted EKF in dynamic localization experiments.

### 4.1. UGV Platform and Hardware Configuration

An unmanned ground vehicle platform equipped with a UWB tag, an IMU module, and incremental wheel encoders was developed for indoor localization experiments. The platform consists of an STM32F103ZET6 onboard controller (Zave, Jinhua, China), an in-house-developed UWB system comprising a vehicle-mounted tag and fixed anchors, an IMU module (WitMotion Shenzhen Co., Ltd., Shenzhen, China), an NRF24L01 wireless control receiver (Your Cee, Shenzhen, China), a remote controller, a TB6612 motor driver (WHEELTEC, Dongguan, China), four drive motors with incremental encoders, a telemetry module, and a battery pack. Together, these components support vehicle control, UWB ranging, yaw-rate measurement, wireless command reception, motor actuation, encoder-based forward-velocity acquisition, data transmission, and power supply.

The STM32F103ZET6 controller serves as the core of the onboard system. It receives motion commands from the NRF24L01 wireless receiver, drives the motors through the TB6612 module, and acquires UWB ranging measurements, IMU yaw-rate data, and encoder pulse counts during vehicle movement. The incremental encoders mounted on the drive motors generate quadrature pulse signals. The STM32 timers operate in encoder mode to count the signed pulse increments and determine the corresponding rotation direction.

The encoder counts are sampled at intervals of Δt=0.1 s, consistent with the localization sampling interval. Let $\Delta c_{l,k}$ denote the pulse-count increment measured by the encoder of the lth wheel at sampling instant k. The encoder-based forward velocity is calculated as

(34) $$v_{\mathrm{enc},k}=\frac{C_{w}}{4\zeta_{\mathrm{enc}}\Delta t}\sum_{l=1}^{4}\alpha_{l}\Delta c_{l,k}$$

where $C_{w}$ is the wheel circumference, $\zeta_{\mathrm{enc}}$ is the effective number of encoder counts per wheel revolution after quadrature decoding. The coefficient $\alpha_{l}\in \{-1,1\}$ corrects the signs of the wheel-encoder increments to match the defined forward direction. The factor of four averages the four wheel-speed measurements. The resulting $v_{\mathrm{enc},k}$ is used as the forward-velocity input $v_{k}$ in the motion model of Section 2.2.

**Figure 4:**
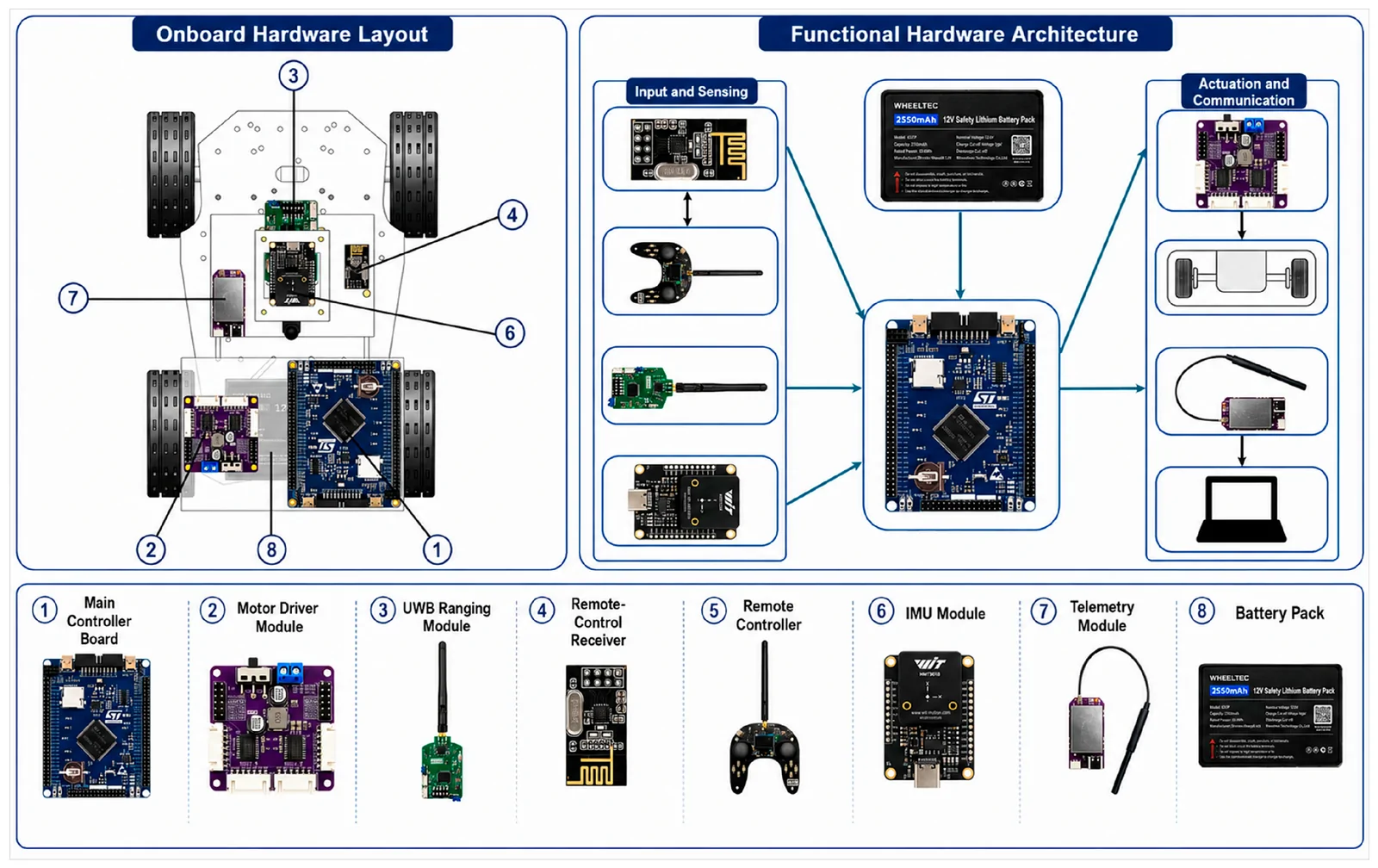
Hardware layout and functional architecture of the UGV platform.

The IMU module provides yaw-rate measurements for heading prediction, while the UWB tag communicates with the fixed anchors and provides range measurements for position correction. The telemetry module transmits experimental data to the external computing unit for recording, visualization, and further algorithm analysis. The battery pack supplies power to the onboard controller, sensing modules, communication modules, and motor driver. The hardware layout and functional connections of the experimental platform are shown in Figure 4.

### 4.2. Software Implementation and Evaluation Flow

**Figure 5:**
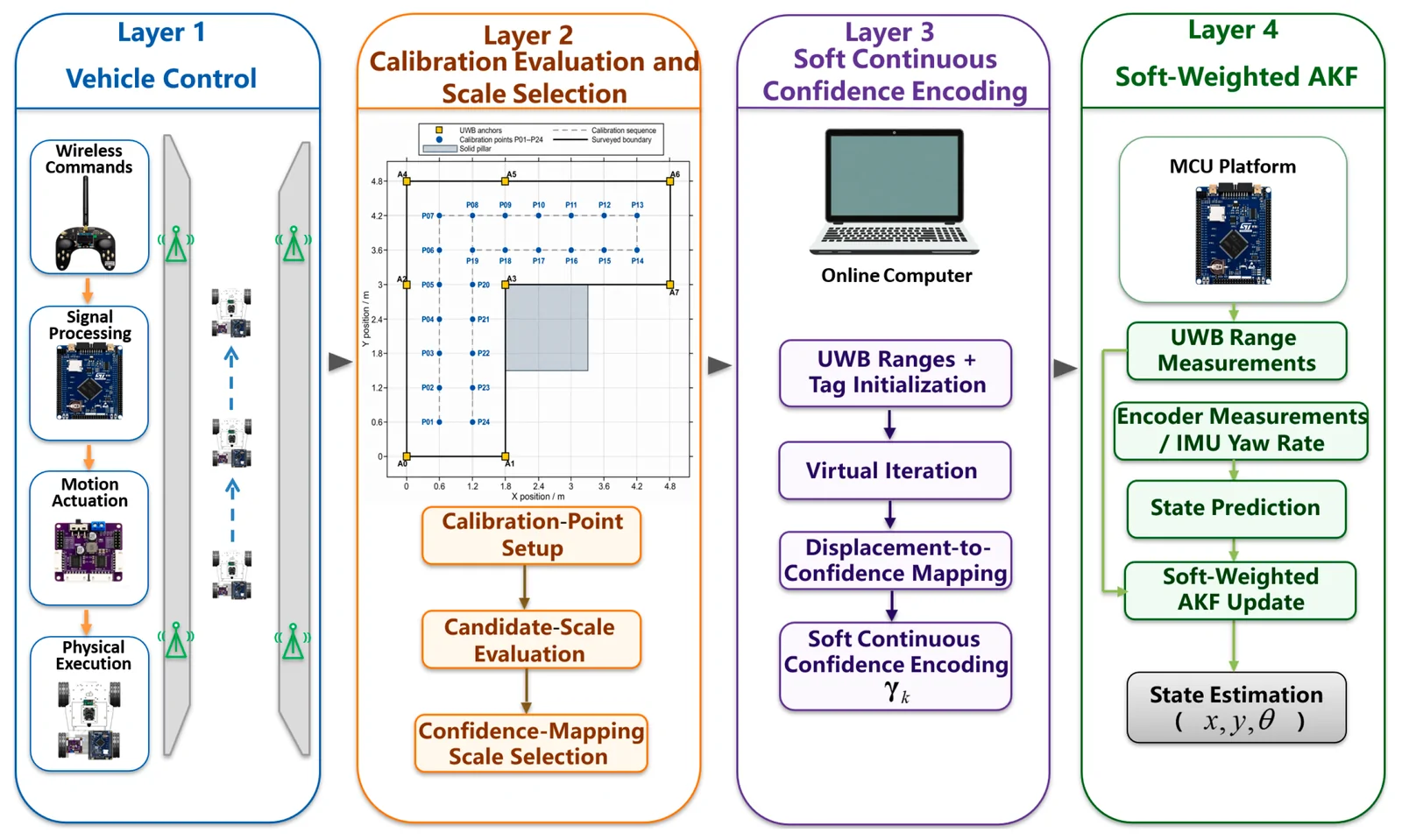
System-level workflow of the soft-weighted AKF localization framework.

The proposed localization framework follows the four-layer workflow shown in Figure 5. Layer 1 performs wireless vehicle control, onboard command processing, motor actuation, and physical execution. Layer 2 uses the surveyed calibration points to evaluate the candidate confidence-mapping scales and selects the scale that provides the best observed positioning performance.

Layer 3 uses the online computer to generate the soft continuous confidence encoding from the current UWB ranges and an available initial tag position. The virtual forward–backward iteration first converts the inconsistency of each range into a terminal virtual-anchor displacement. These displacements are then mapped to continuous confidence coefficients using the selected confidence-mapping scale. The resulting coefficient vector $\gamma_{k}$ represents the soft continuous confidence encoding.

Layer 4 incorporates soft encoding into the AKF update. The vehicle state is estimated using the motion model, while the measurement-noise covariance is adaptively updated during localization. The forgetting factor for the adaptive covariance update in the AKF was set to β=0.97. The corresponding confidence coefficients regulate the contributions of the individual UWB measurements.

### 4.3. Experimental Evaluation of Soft Continuous Confidence Encoding and Weighting

#### 4.3.1. Calibration-Site Evaluation and Confidence-Mapping Scale Selection

**Figure 6:**
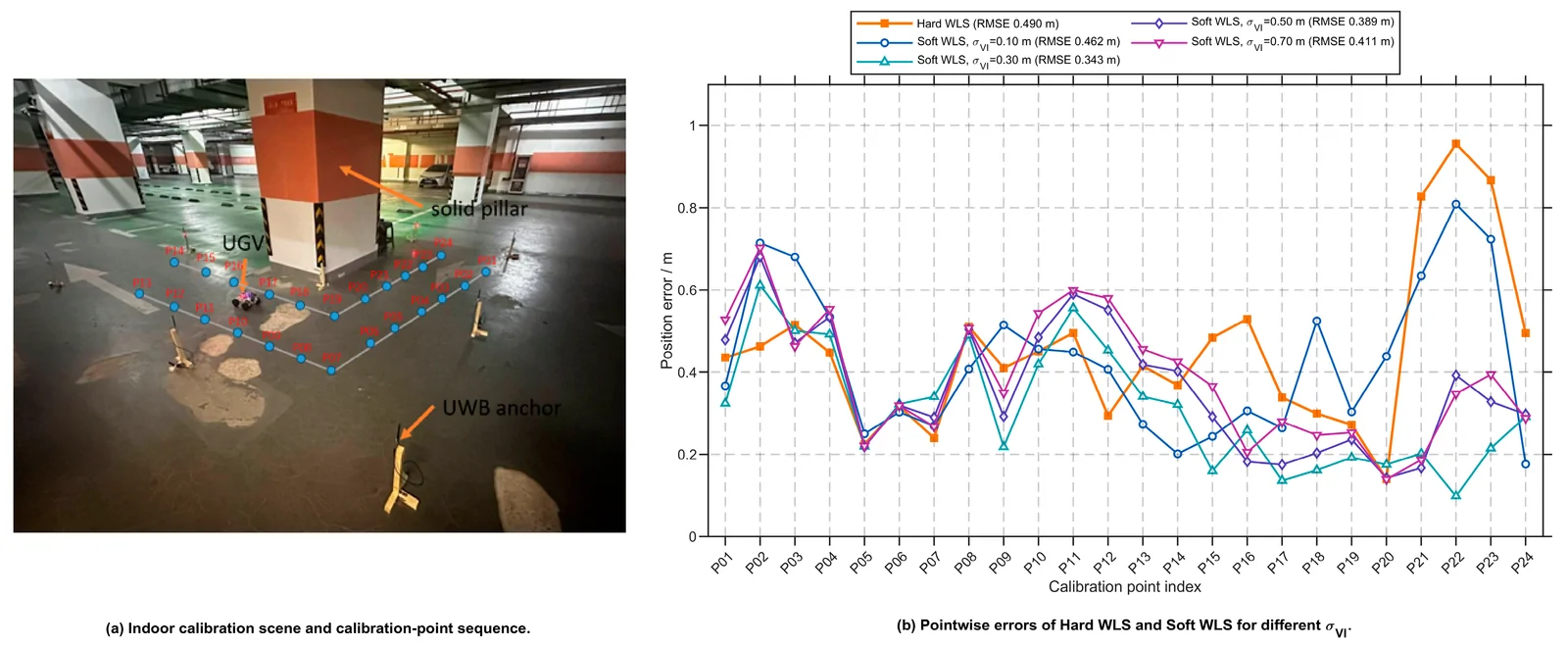
Calibration-site evaluation and confidence-mapping scale selection. (**a**) Indoor experimental scene and calibration-point sequence P01–P24. (**b**) Pointwise position errors of Hard WLS and Soft WLS for $\sigma_{\mathrm{VI}}$ = 0.10, 0.30, 0.50, and 0.70 m.

Figure 6a shows the indoor experimental scene and the calibration-point sequence P01–P24. Figure 2 presents the surveyed layout of anchors A0–A7, the 1.5 m × 1.5 m solid pillar, and the calibration points. The calibration points were arranged on a 0.6 m grid within the surveyed L-shaped area. Their surveyed coordinates served as the ground-truth positions.

At each calibration point, hard encoding and soft encoding were separately incorporated into the same WLS objective in Equation (13). Hard WLS used the map-derived hard encoding at the surveyed point, whereas Soft WLS used the soft encoding obtained using the method described in Section 3.2.

As shown in Figure 6b and Table 1, Hard WLS produced an RMSE of 0.490 m. The Soft WLS RMSEs were 0.462, 0.343, 0.389, and 0.411 m for $\sigma_{\mathrm{VI}}$ = 0.10, 0.30, 0.50, and 0.70 m, respectively. All four Soft WLS configurations achieved lower RMSEs than Hard WLS. Among the tested confidence-mapping scales, $\sigma_{\mathrm{VI}}$ = 0.30 m yielded the lowest RMSE, corresponding to a reduction of approximately 30%. It also produced the lowest MAE, 95th-percentile error, and maximum error.

The improvement was particularly evident in the suppression of large positioning errors. Compared with Hard WLS, Soft WLS with $\sigma_{\mathrm{VI}}$ = 0.3 m reduced the 95th-percentile error from 0.861 to 0.548 m and the maximum error from 0.956 to 0.611 m. The pointwise curves in Figure 6b also show that Soft WLS substantially reduced the large error peaks observed for Hard WLS at several calibration points.

Under hard encoding, an anchor range is either retained or excluded, whereas soft encoding preserves continuous differences in the geometric consistency of individual UWB ranges. Consequently, a partially reliable range can still contribute to the WLS solution, while a range with lower confidence is assigned less influence in the optimization. The lower aggregate and tail errors demonstrate that soft weighting provides a more reasonable and precise allocation of individual anchor contributions under the evaluated conditions.

The comparison among the four candidate values shows that the confidence-mapping scale affects the positioning performance of Soft WLS. Because $\sigma_{\mathrm{VI}}$ = 0.3 m provided the best overall performance, it was adopted for the subsequent UGV localization experiments in Section 4.3.2.

#### 4.3.2. Comparison of Localization with/Without AKF

**Figure 7:**
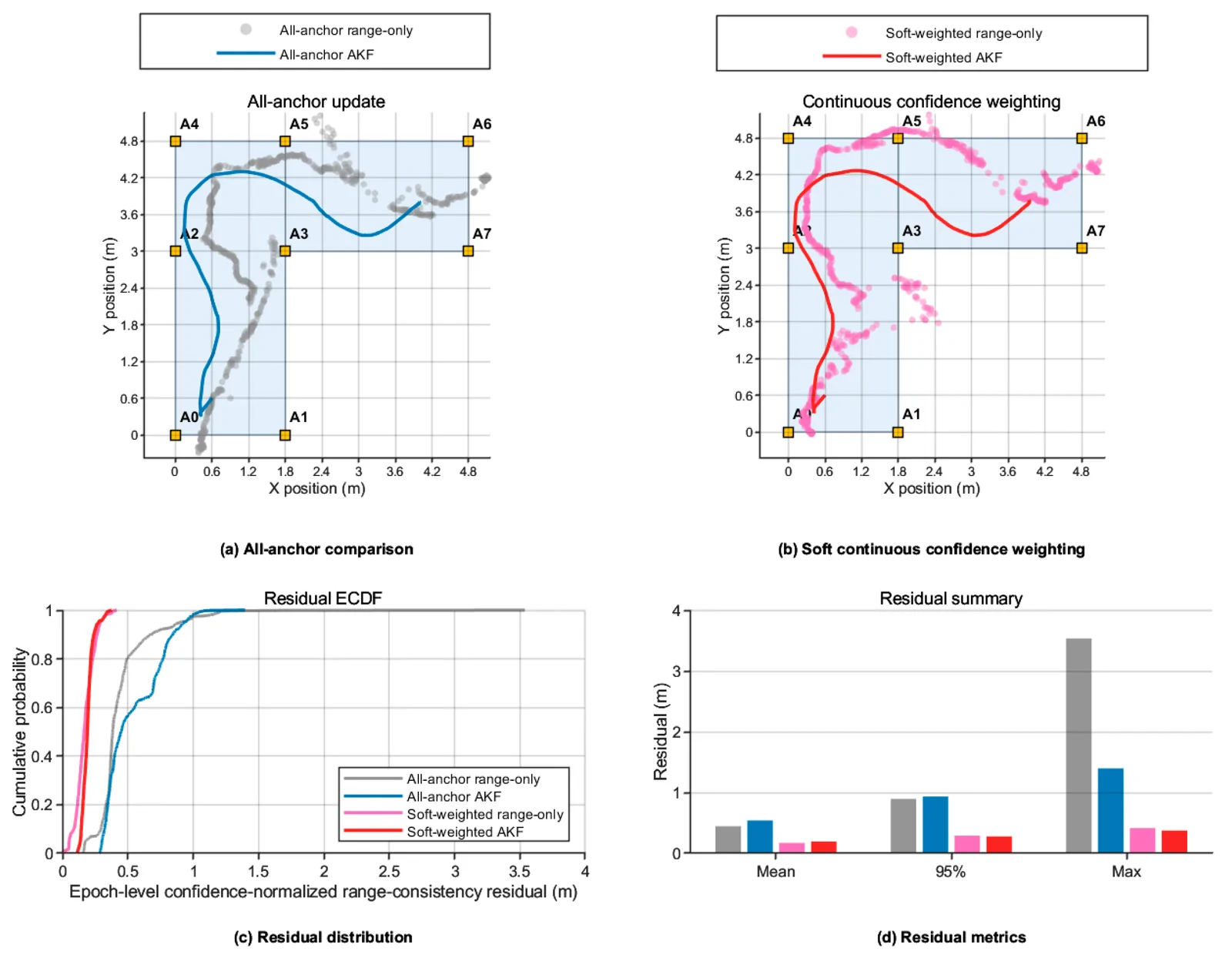
Comparative evaluation of localization with and without AKF. (**a**) All-anchor range-only output and all-anchor AKF trajectory. (**b**) Soft-weighted range-only output and soft-weighted AKF trajectory. (**c**) ECDFs of the epoch-level confidence-normalized mean absolute range-consistency residuals. (**d**) Mean, 95th-percentile, and maximum residuals for the four outputs.

Figure 7 compares the localization results with and without AKF under the all-anchor and soft continuous confidence-weighting configurations. Panels (a) and (b) show the all-anchor and soft-weighted results, respectively, and each panel compares the range-only output with the corresponding AKF trajectory. In both configurations, the range-only outputs contain pronounced epoch-to-epoch jumps, whereas AKF produces substantially more continuous trajectories. This shows that incorporating encoder/IMU information effectively suppresses abrupt position updates.

Table 2 quantitatively confirms the improvement in trajectory continuity. After AKF fusion, the P95 step length decreased to 0.016 m in both configurations. The maximum step length decreased from 3.509 to 0.322 m for the all-anchor configuration and from 4.131 to 0.263 m for the soft-weighted configuration. However, the mean residual did not decrease after AKF fusion, indicating that AKF mainly constrains abrupt UWB-based position changes rather than minimizing the instantaneous range residual.

Figure 7c,d additionally show that the soft-weighted outputs have a narrower residual distribution than the all-anchor outputs. Nevertheless, the soft-weighted range-only output retains large position jumps despite its low residuals. Thus, soft weighting improves the reliability of the UWB measurement input, whereas AKF is primarily responsible for improving temporal continuity.

**Figure 8:**
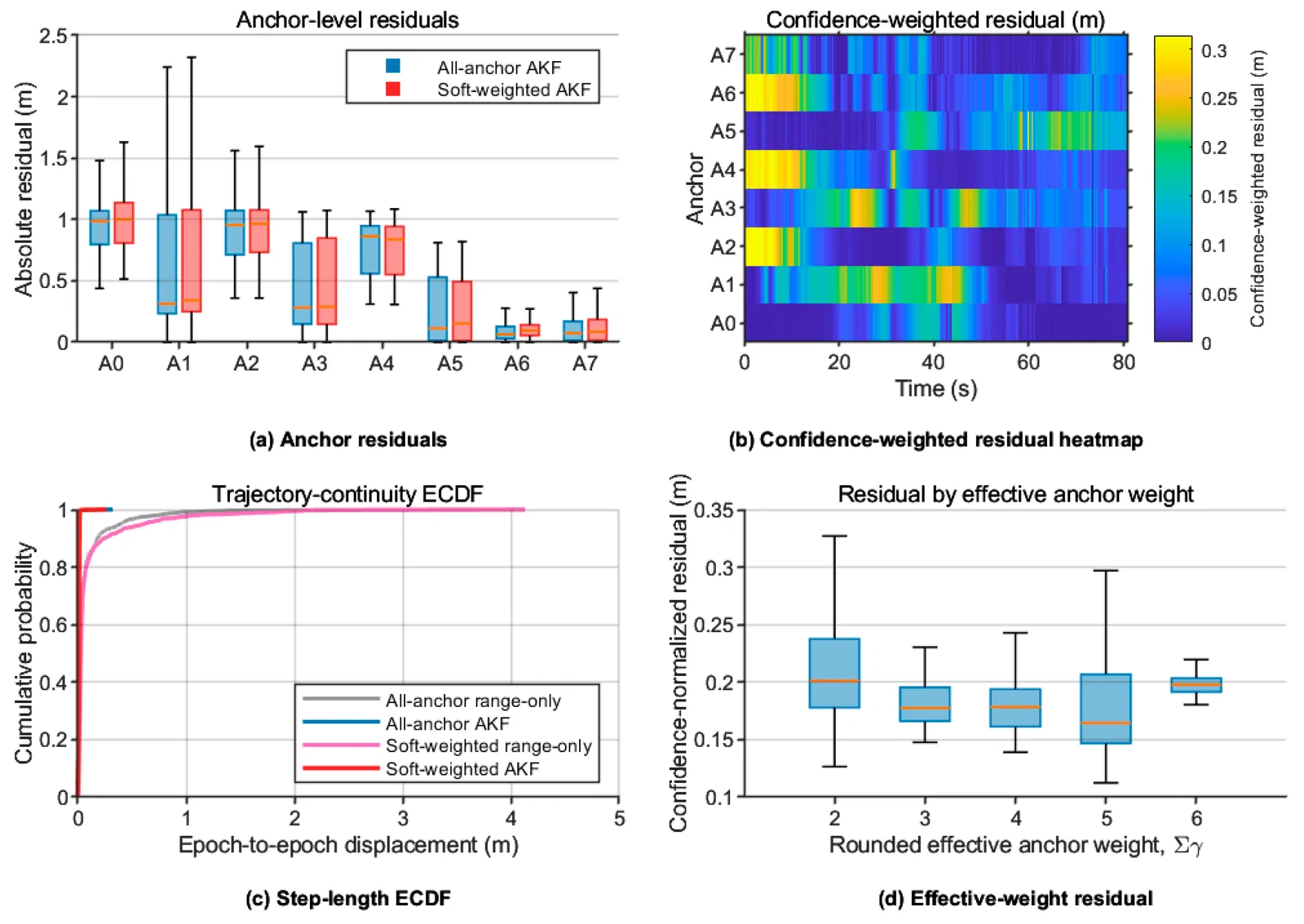
Anchor-level residual and trajectory-continuity analysis. (**a**) Anchor-wise absolute range residual distributions for the all-anchor AKF and soft-weighted AKF. (**b**) Time-resolved confidence-weighted residuals for soft-weighted AKF. (**c**) ECDFs of epoch-to-epoch displacement for the four outputs. (**d**) Confidence-normalized residuals of soft-weighted AKF grouped by rounded effective anchor weight.

Figure 8 further supports the effect of AKF on trajectory continuity. In Figure 8c, the step-length distributions of both AKF outputs are concentrated near zero, whereas the corresponding range-only outputs exhibit long tails. Figure 8a,b,d further show that the residual characteristics differ among anchors and vary with time and effective anchor weight, supporting the use of continuous confidence weighting for the UWB measurement updates.

**Figure 9:**
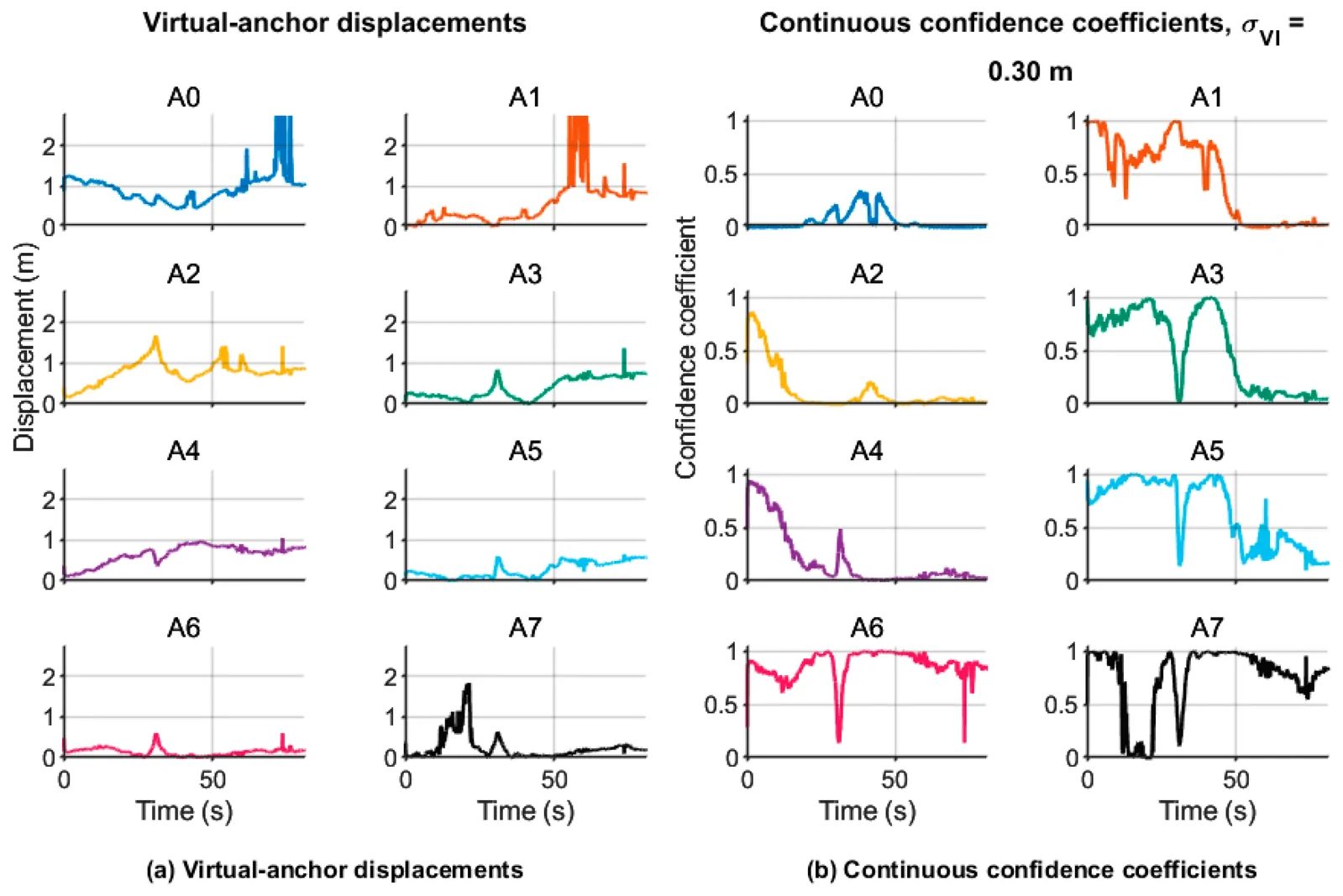
Virtual-anchor displacements and continuous confidence coefficients. (**a**) Virtual-anchor displacements for anchors A0–A7. (**b**) Corresponding continuous confidence coefficients for $\sigma_{\mathrm{VI}}$ = 0.30 m.

Figure 9 plots the virtual-anchor displacements and corresponding continuous confidence coefficients for anchors A0–A7. Larger virtual-anchor displacements generally corresponded to lower confidence coefficients. The continuous variation of these coefficients reflects the time-varying confidence assigned to each UWB range.

Overall, AKF primarily suppresses abrupt epoch-to-epoch position changes and improves trajectory continuity, while soft continuous confidence weighting reduces the influence of low-confidence UWB ranges. Their combination therefore provides more reliable measurement updates and a continuous localization trajectory.

#### 4.3.3. Comparison and Analysis of Soft-Weighted AKF and Hard-Weighted EKF

An AKF was incorporated into the soft continuous confidence-weighted localization method, and its results were compared with those of a hard binary confidence-weighted EKF implemented according to the confidence-encoding strategy in [17].

As shown in Table 3, Soft-weighted AKF achieved mean, P95, and maximum residuals of 0.193, 0.278, and 0.375 m, respectively, compared with 0.335, 0.430, and 0.510 m for Hard-weighted EKF. Hard-weighted EKF retained an average of 6.39 input links. Soft-weighted AKF used eight valid input links, with an average effective weight of 3.60 and a mean confidence coefficient of 0.450. The two methods produced similar P95 epoch-to-epoch displacements of 0.019 and 0.016 m, respectively.

**Figure 10:**
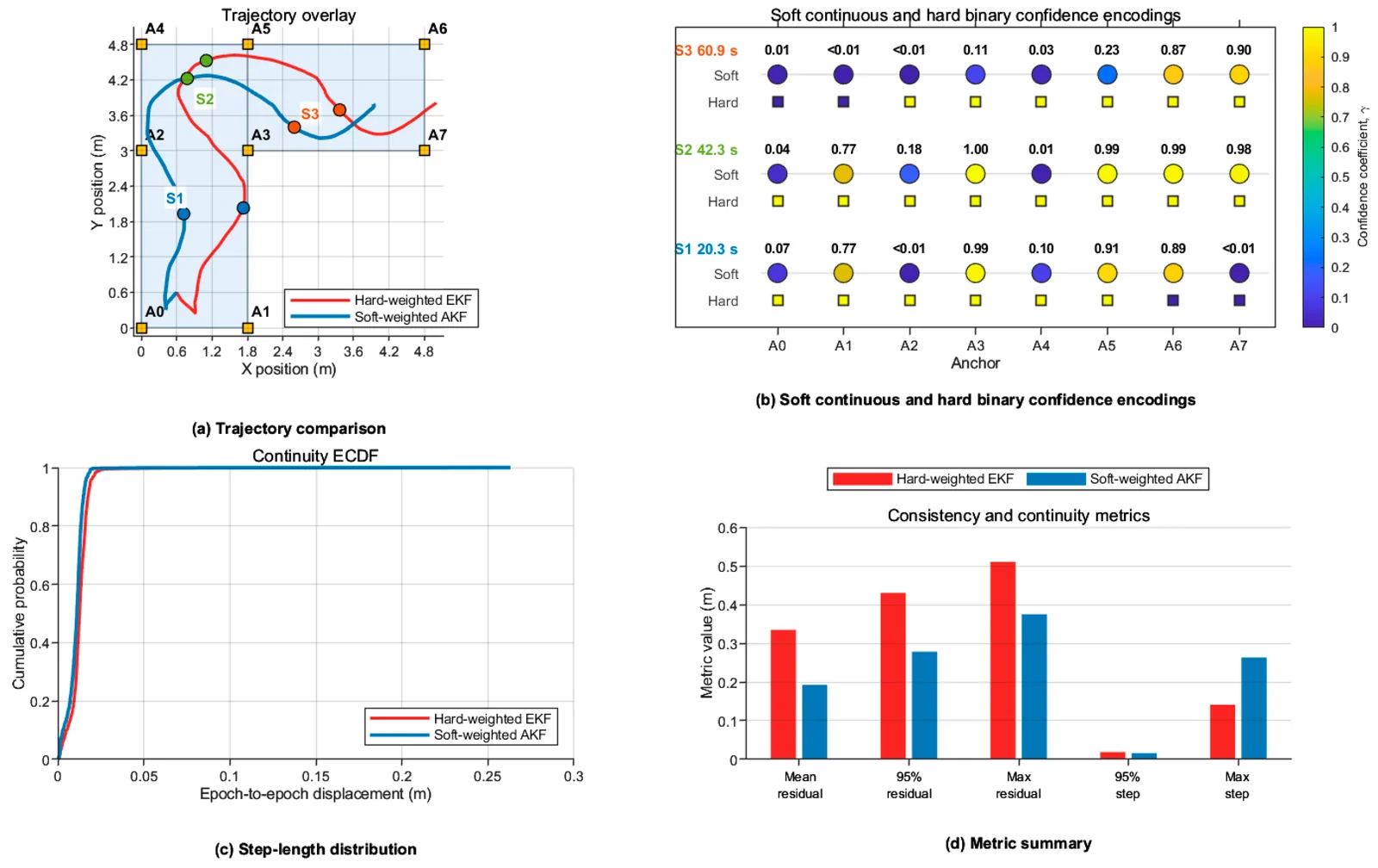
Comparison of Hard-weighted EKF and Soft-weighted AKF. (**a**) Trajectory comparison. (**b**) Soft continuous and hard binary confidence encodings at selected epochs. (**c**) ECDFs of epoch-to-epoch displacement. (**d**) Summary of weighted range-consistency and trajectory-continuity metrics.

Figure 10a shows differences between the trajectories obtained using the two filtering configurations. Figure 10b presents the confidence encodings at epochs S1–S3, showing the different anchor contributions produced by hard encoding and soft encoding. The step-length ECDFs in Figure 10c are close over most of their distributions, while Figure 10d summarizes the differences in residual and displacement metrics.

**Figure 11:**
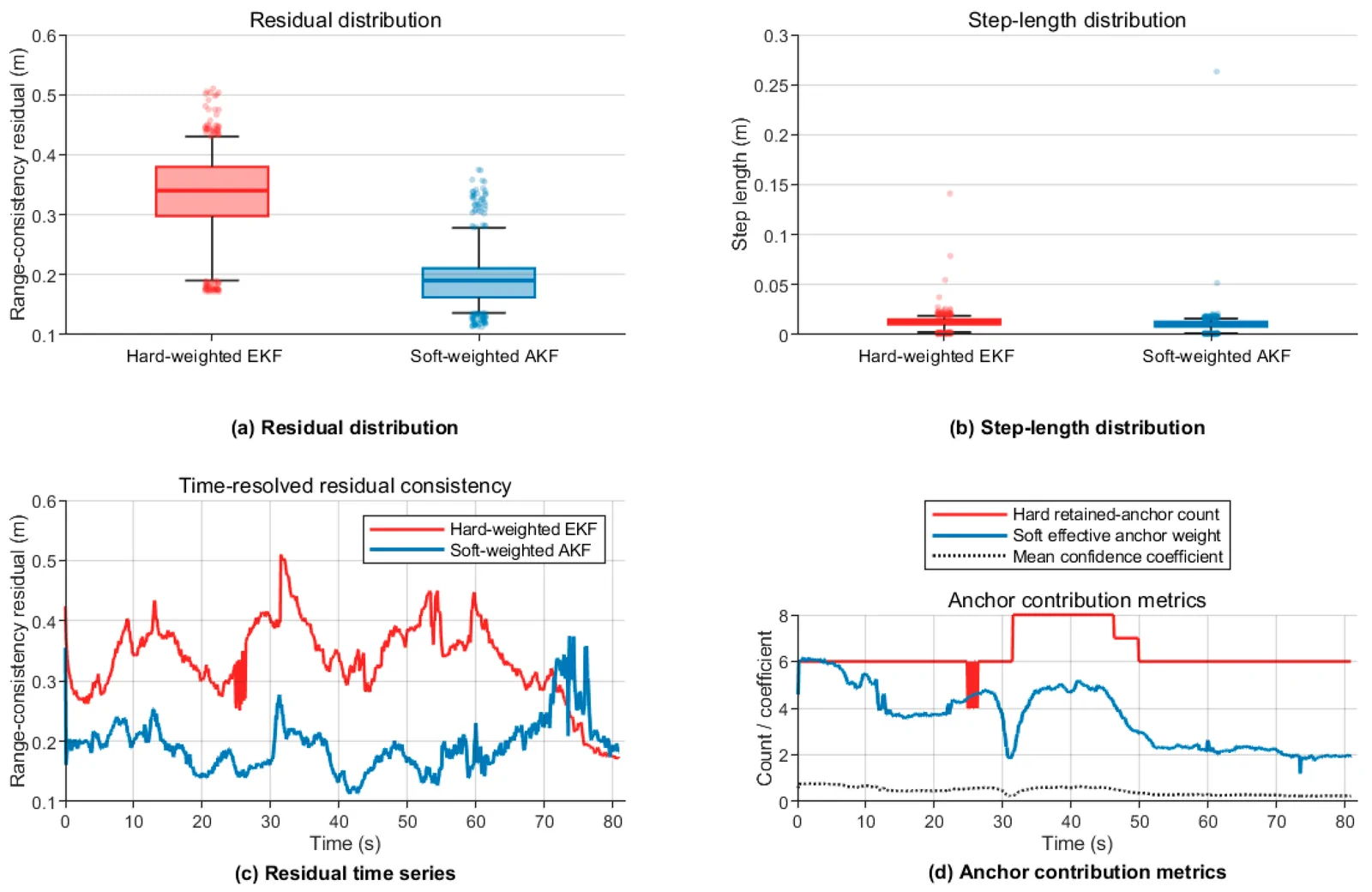
Residual and trajectory-continuity analysis of Hard-weighted EKF and Soft-weighted AKF. (**a**) Weighted range-consistency residual distribution. (**b**) Epoch-to-epoch displacement distribution. (**c**) Time-resolved residual consistency. (**d**) Anchor-contribution metrics over time.

Figure 11 provides a more detailed comparison of the residual and displacement characteristics. Figure 11a shows that the residuals of Soft-weighted AKF were concentrated within a lower range. Figure 11b shows that both methods exhibited small step lengths during most localization epochs, with only a few larger displacements. The time-resolved results in Figure 11c show that Soft-weighted AKF maintained lower residuals over most of the sequence. Figure 11d presents the temporal variations in the retained-anchor count, effective anchor weight, and mean confidence coefficient.

#### 4.3.4. Comparison with Variational Bayesian and RANSAC-Based Baselines

To broaden the baseline evaluation beyond hard binary confidence encoding, the proposed Soft-Weighted AKF was further compared with a variational Bayesian EKF (VB-EKF) and a RANSAC-based AKF (RANSAC-AKF). VB-EKF represents continuous adaptation of measurement uncertainty, whereas RANSAC-AKF uses consensus-based rejection of inconsistent UWB ranges. Performance was assessed using a common geometric-residual criterion across all valid UWB links.

As shown in Table 4, Soft-Weighted AKF achieved the lowest mean absolute, RMS, and P95 residuals, demonstrating the best overall range–trajectory consistency among the three methods. Its advantage was most evident in the upper tail, with a 20.7% lower P95 residual than RANSAC-AKF and a slightly lower P95 residual than VB-EKF. Although VB-EKF yielded the lowest median residual, Soft-Weighted AKF more effectively limited large residuals, indicating greater robustness to persistently inconsistent UWB measurements.

**Figure 12:**
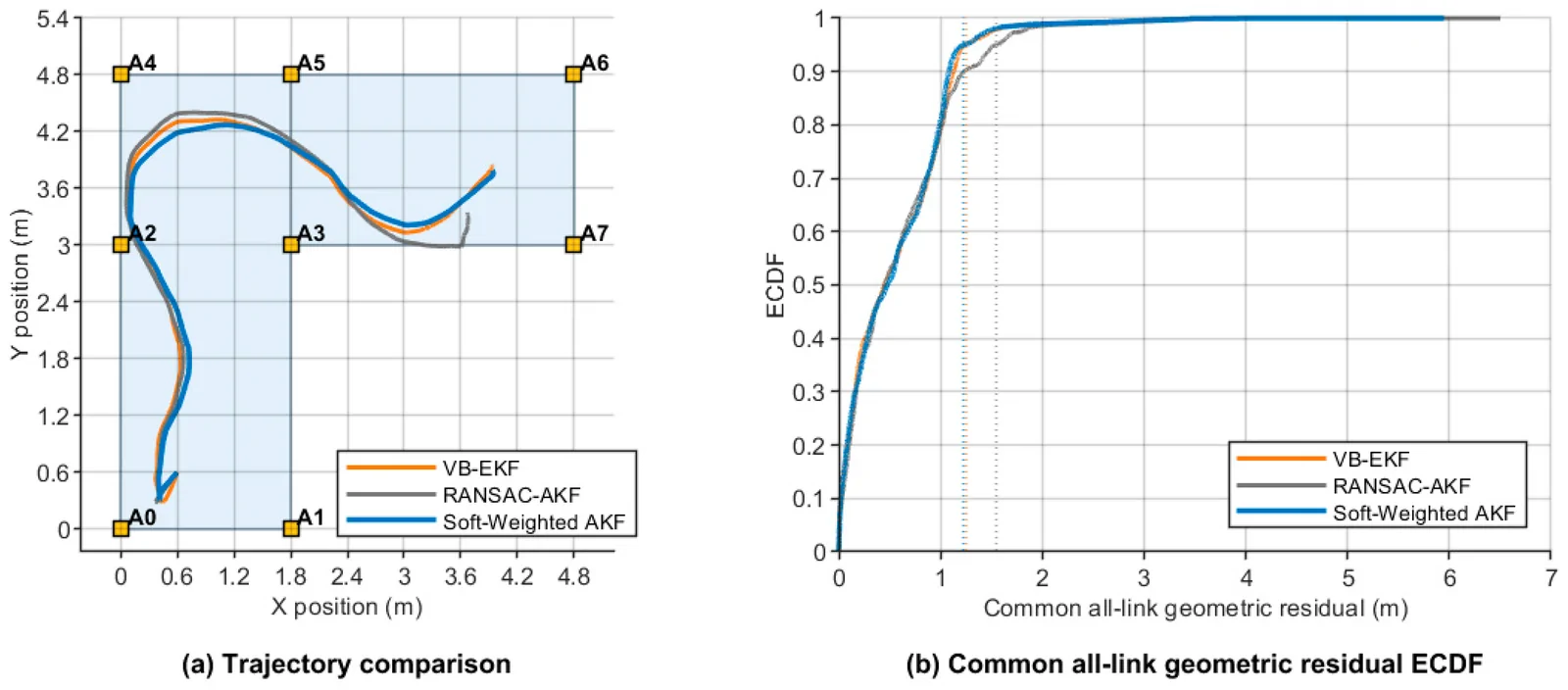
Comparison of VB-EKF, RANSAC-AKF, and Soft-Weighted AKF. (**a**) Estimated trajectories in the surveyed L-shaped environment. (**b**) Empirical cumulative distribution functions (ECDFs) of the common all-link geometric residuals; the vertical dotted lines indicate the corresponding P95 values.

Figure 12a shows that all three methods preserved the overall L-shaped trajectory. However, RANSAC-AKF exhibited greater divergence along the upper and right-hand segments, together with a more pronounced terminal deviation. The ECDFs in Figure 12b largely overlapped within the central residual range but diverged in the upper tail. Soft-Weighted AKF exhibited the lowest P95 residual, indicating stronger suppression of large geometric inconsistencies.

Taken together, these results show that continuous confidence weighting provides a more balanced treatment of uncertain UWB links than the two baseline strategies. Soft-Weighted AKF preserved the overall trajectory structure while achieving the lowest mean, RMS, and P95 residuals, demonstrating improved geometric consistency and residual-tail robustness.

### 4.4. Online Computation and Computational Cost

In the current experimental system, the UGV exchanges data with the online computer through the telemetry module. The UWB ranges, encoder pulse increments, and IMU yaw-rate measurements are transmitted to the online computer. The virtual forward-backward iteration, soft continuous confidence calculation, and AKF state estimation are then performed online. The resulting localization state is returned to the UGV through the same telemetry link. This communication and computation process operated continuously during UGV motion throughout the experiments.

To quantify the computational cost of the core virtual forward-backward iteration, its execution time and convergence behavior were evaluated using the calibration-site and dynamic-trajectory datasets described in Section 4.3.1, Section 4.3.2, Section 4.3.3 and Section 4.3.4. The procedure was executed once for each valid UWB range frame or localization epoch. One complete iteration comprised one reverse anchor update followed by one forward tag update. Table 5 reports the measurements obtained on the online computer used in the experiments.

For the calibration-site experiment, the iteration was evaluated over 6861 valid UWB range frames. The mean time per iteration was 0.084 ms, and convergence required 2.614 iterations on average. In total, 97.3% of the frames converged within two or three iterations, and all frames converged within four iterations. The estimated mean computation time of the iterative procedure was therefore approximately 0.220 ms per frame.

For the dynamic trajectory, the iteration was evaluated over 810 localization epochs. The mean time per iteration was 0.065 ms, and convergence required 2.985 iterations on average. Overall, 92.1% of the epochs converged within two or three iterations, and all epochs converged within five iterations. The estimated mean computation time was therefore approximately 0.194 ms per epoch.

Overall, the virtual forward–backward iteration converged within five iterations for all evaluated samples. The estimated mean computation time of the iterative procedure remained below 0.23 ms per frame or localization epoch for both datasets.

## 5. Conclusions

This study developed a UWB/IMU localization framework that uses soft continuous confidence encoding and weighting for UGVs operating in structured indoor environments under mixed LOS/NLOS propagation. The principal contribution is the integration of two complementary mechanisms. Soft encoding represents continuous differences in the geometric consistency of individual UWB ranges, whereas the AKF accommodates temporal variations in measurement uncertainty through online covariance adaptation. This integration retains partially reliable ranges in localization while limiting the influence of measurements that are inconsistent with the current tag–anchor geometry. The calibration and dynamic experiments demonstrate that soft weighting improves the allocation of anchor contributions, whereas AKF fusion primarily enhances trajectory continuity. These findings indicate that measurement-reliability modeling and temporal state estimation address complementary aspects of localization degradation. Unlike hard encoding, the proposed method directly obtains the confidence coefficients at the current vehicle position without determining the current code from neighboring points.

Nevertheless, the present evaluation is limited to a single surveyed L-shaped environment, a fixed eight-anchor deployment, and one UGV platform. Although this setting captures corridor-like passages, corners, and LOS/NLOS transitions representative of structured indoor environments, it cannot fully reflect the spatial scale, layout diversity, and operating complexity of practical deployments. Future studies will therefore evaluate the proposed framework in larger and more diverse environments with varied layouts, anchor geometries, vehicle speeds, and dynamic occlusion conditions. These evaluations will further examine its robustness, generalizability, and localization continuity under more challenging operating conditions. Future work will also investigate online adaptation of the confidence-mapping scale under changing propagation conditions. The complete localization framework will be implemented on an onboard embedded platform to further evaluate end-to-end latency, processor utilization, and memory consumption. Collectively, these efforts will help clarify the practical deployment boundaries of the proposed framework and support its application to more complex indoor navigation scenarios.

## Figures and Tables

**Table 1:** Calibration-point position-error metrics for Hard WLS and Soft WLS under the four confidence-mapping scales.

Method	$\sigma_{\mathrm{VI}}$ (m)	RMSE (m)	MAE (m)	P95 (m)	Max (m)	RMSE Reduction (%)
Hard WLS	—	0.490	0.450	0.861	0.956	—
Soft WLS	0.10	0.462	0.427	0.722	0.809	5.6
**Soft WLS**	**0.30**	**0.343**	**0.312**	**0.548**	**0.611**	**30.0**
Soft WLS	0.50	0.389	0.360	0.584	0.680	20.5
Soft WLS	0.70	0.411	0.384	0.596	0.703	16.0

Note: Metrics were calculated from the 24 pointwise errors. P95 denotes the empirical 95th-percentile error. RMSE reduction was calculated relative to Hard WLS. The best-performing configuration among the evaluated settings is shown in bold.

**Table 2:** Quantitative comparison of localization with and without AKF.

Configuration	Residual (m) Mean/P95/Max	Mean Effective Anchor Weight	Step Length (m) P95/Max
All-anchor range-only	0.446/0.896/3.537	8.00	0.394/3.509
All-anchor AKF	0.541/0.933/1.397	8.00	0.016/0.322
Soft-weighted range-only	0.171/0.291/0.415	3.89	0.608/4.131
Soft-weighted AKF	0.193/0.278/0.375	3.60	0.016/0.263

Note: Residual denotes the epoch-level confidence-normalized mean absolute range-consistency residual. Mean effective anchor weight denotes the epoch-averaged sum of the confidence coefficients. P95 denotes the empirical 95th percentile.

**Table 3:** Quantitative comparison of Hard-weighted EKF and Soft-weighted AKF.

Configuration	Weighted Residual (m) Mean/P95/Max	Link Contribution Input/Effective/Mean Confidence	Epoch-to-Epoch Displacement (m) P95/Max
Hard-weighted EKF	0.335/0.430/0.510	6.39/6.39/1.000	0.019/0.141
Soft-weighted AKF	0.193/0.278/0.375	8.00/3.60/0.450	0.016/0.263

Note: Weighted residual denotes the confidence-weighted mean absolute range-consistency residual, and P95 denotes the empirical 95th percentile. In the link-contribution column, input denotes the mean number of input links, effective denotes the mean effective anchor weight, and mean confidence denotes the effective weight divided by the number of input links. For Hard-weighted EKF, the input links are the anchors retained by hard encoding; for Soft-weighted AKF, they are the valid UWB measurements.

**Table 4:** Comparison of common all-link geometric residuals for VB-EKF, RANSAC-AKF, and Soft-Weighted AKF.

Method	Mean Absolute Residual (m)	RMS Residual (m)	Median Residual (m)	P95 Residual (m)
VB-EKF	0.558	0.756	0.447	1.256
RANSAC-AKF	0.588	0.806	0.452	1.547
Soft-Weighted AKF	0.554	0.743	0.479	1.227

Note: Residuals are unweighted absolute differences between the measured and position-implied UWB ranges, evaluated over the same valid links for all methods. P95 denotes the empirical 95th percentile.

**Table 5:** Computational metrics of the virtual forward-backward iteration.

Experiment	Sample Count	Mean Time per Iteration (ms)	Mean Iterations to Convergence	Iteration Distribution
Calibration site	6861	0.084	2.614	2: 2828 (41.2%); 3: 3851 (56.1%); 4: 182 (2.7%)
Dynamic trajectory	810	0.065	2.985	2: 98 (12.1%); 3: 648 (80.0%); 4: 42 (5.2%); 5: 22 (2.7%)

Note: Sample count denotes the number of valid UWB range frames in the calibration-site experiment and the number of localization epochs in the dynamic-trajectory experiment. Mean time per iteration is the average time required for one complete iteration. Mean iterations to convergence is the average number of complete iterations required to satisfy the convergence criterion. Each iteration-distribution entry is formatted as iterations to convergence: sample count (percentage within the corresponding experiment). The reported times cover only the virtual forward-backward iteration and exclude data loading, confidence mapping, AKF state estimation, telemetry communication, and output rendering.

## Data Availability

Data will be made available on request.

## Abbreviations

The following abbreviations are used in this manuscript:

AKF	Adaptive Kalman filter
DS-TWR	Double-sided two-way ranging
ECDF	Empirical cumulative distribution function
EKF	Extended Kalman filter
ESKF	Error-state Kalman filter
EULF-IUB	Enhanced UGV localization framework using tightly coupled IMU/UWB/barometer integration
GNSS	Global Navigation Satellite System
IMU	Inertial measurement unit
INS	Inertial navigation system
LED	Light-emitting diode
LiDAR	Light detection and ranging
LOS	Line-of-sight
MAE	Mean absolute error
MEMS	Microelectromechanical systems
MR-ULINS	Tightly coupled UWB–LiDAR–inertial estimator with multi-epoch outlier rejection
NLOS	Non-line-of-sight
RANSAC	Random sample consensus
RMSE	Root mean square error
P95	95th percentile
SLAM	Simultaneous localization and mapping
ToF	Time-of-flight
UAV	Unmanned aerial vehicle
UGV	Unmanned ground vehicle
UWB	Ultra-wideband
VIO	Visual–inertial odometry
VLC	Visible light communication
VLP	Visible light positioning
WLS	Weighted least squares
